# Prognostic variables for conditional survival in patients with esophageal squamous cell carcinoma who underwent minimally invasive surgery

**DOI:** 10.1186/s12885-022-09423-5

**Published:** 2022-03-26

**Authors:** Mingqiang Liang, Jiazhou Xiao, Maohui Chen, Bin Zheng, Chun Chen

**Affiliations:** 1 Department of Thoracic Surgery, Fujian Medical University Union Hospital, No.29 Xinquan Road, Fuzhou, 350001 Fujian China; 2 Key Laboratory of Cardio-Thoracic Surgery (Fujian Medical University), Fujian Province University, Fuzhou, 350001 Fujian China

**Keywords:** Conditional survival, Esophageal squamous cell carcinoma, Minimally invasive surgery, Prognosis

## Abstract

**Background:**

Esophageal squamous cell carcinoma (ESCC) survival is mainly reported at the time of treatment. Conditional survival is another prognostic tool to evaluate ESCC patients who has survived more than one year since treatment.

**Methods:**

We analyzed data from 705 ESCC patients who underwent minimally invasive surgery between 2013 and 2016. Using the Kaplan–Meier method, we computed a 5-year relative conditional survival. We also investigated the prognostic factors associated with survival using Cox proportional hazards models.

**Results:**

Conditional survival improved over time for all cohorts of ESCC patients who survived a period after surgery. The greatest improve in conditional survival were observed in patients 2 years after surgery. In addition, the results of the Cox survival model from the time of surgery, T stage (*p* < 0.001), N stage (*p* < 0.001), and anastomotic leak (*p* = 0.022), were significantly associated with survival. However, the results of the Cox survival model from 2 years after surgery, N stage (*p* < 0.001), and anastomotic leak (*p* = 0.032) were significantly associated with survival.

**Conclusion:**

For ESCC patients who survived a period after surgery, the largest increases in conditional survival were observed in patients 2 years after surgery. We suggest that patients with anastomotic leakage and higher T and N stages should be strictly screened according to various time, and that conditional survival should be used as a powerful prognostic tool for ESCC patients.

## Background

Esophageal cancer is the seven most frequent tumor malignancy characterized by high morbidity and is ranked sixth in cancer-related deaths worldwide [1]. There are two major histological types: esophageal squamous cell carcinoma (ESCC) and esophageal adenocarcinoma (EA) [2]. The former frequently occur in Asian countries, particularly in China, where it accounts for more than 90% of total cases, while the latter is more predominant in western countries [3].

Prognosis is an important issue for ESCC patients and their oncologists [4]. Traditional prognostic assessments are mainly focused on survival rates of a specific time, such as the 5 years survival rate. However, these tools are constant, and cannot provide accurate prognostic evaluations, especially when patients have lived a long time after surgery [5]. For example, it is obvious that patients with ESCC who live more than 2 years represent a group with much better prognosis for continuing survival in clinical practice, but this phenomenon has not been clearly testified.

Conditional survival is the probability of surviving into the forthcoming years based on previous survival to a specified time point [6]. To the best of our knowledge, several studies specifically examined the conditional survival of patients with esophageal cancer. Using the Surveillance, Epidemiology, and End Results (SEER) database, Deng and colleagues [7] published a large-scale examination of conditional survival in esophageal or gastroesophageal junction cancer by evaluating 25,232 patients who were diagnosed between 2000 and 2016. Shin and colleagues [8] evaluated conditional survival in a better-characterized group of patients who underwent complete resection for ESCC at a tertiary cancer center in Korea from 1994 to 2016. Hagens and colleagues [9] reported conditional survival after neoadjuvant chemoradiotherapy and surgery for esophageal cancer in the Netherlands from 2004 to 2019.

However, all these studies involved patients over a period of more than 10 years, during which, the strategy for treating esophageal cancer changed [10–12]. Existing evidence suggests that the prognosis of esophageal cancer patients, heavily depends on the treatment modalities [13, 14], which would undermine the credibility of the study. Hence, we used our database to evaluate conditional survival in the patients from 2013 to 2016. The purpose of the present study was to define conditional survival among ESCC patients undergoing minimally invasive surgery, and to identify the factors influencing survival after surgery.

## Methods

### Study population

The study included all patients who received minimally invasive surgery for esophageal squamous cell carcinoma in Fujian Medical University Union Hospital between January 2013 and December 2016 (*N* = 811). It excluded patients who had history of other cancers before esophageal cancer diagnosis (*N* = 27), received neoadjuvant chemotherapy or radiotherapy before surgery (*N* = 65), and who did not have R0 resection (*N* = 14). This resulted in 705 patients for final analyses. This study proposal was approved by the Institutional Review Board of our hospital, which waived the need for informed consent from each individual patient for its retrospective design.

### Data collection and follow-up

Data were obtained from electronic medical records (EMR), including age, sex, tumor location, tumor grade, pathologic stage (TNM classification and AJCC 8^th^ edition staging), and treatment related factors about surgery such as anastomotic leak, pulmonary infection, chylothorax, and adjuvant therapy.

The program of the postoperative follow-ups was carried out as below: every three months for the first two years, every six months from the third to fourth year, and 12 months thereafter. The routine follow-up schedules included a physical examination, laboratory test, and thoracic and abdominal computed tomography. If recurrence was suspected, an additional workup was performed with PET/CT. All patients were follow-upped until death or final date in December 2020. Overall survival (OS) in this study was defined as the time from surgery to death for any reason.

### Outcomes and statistical analysis

The study primary outcome was associated with conditional survival (CS) from various time points after surgery. Conditional survival was computed from traditional Kaplan–Meier data [15]. The arithmetic definition of CS can be stated as follows: CS(y|x) = CS(y + x)/CS(x). It is the likelihood of surviving an additional y years, assumed that the person has already survived x years [15]. For example, to measure the 3-year CS for a patient who already survived 2 years, CS (3 + 2) is calculated from the survival at 5 years divided by the survival at 2 years. When a survival curve has an altering hazard rate over time, this will be revealed as a alteration in CS as more time passes from the time of surgery.

In this study, conditional survival points for selected survival durations were calculated from the time of surgery until 5 years after surgery. An additional conditional survival analysis was performed with the patients’ population that was divided into 3 groups based on TNM staging: Stage I, Stage II, and Stage III/IV.

The study secondary outcome was related with the identification of factors associated with survival using Cox proportional hazards (PH) models. A univariate Cox PH model was used to individually evaluate each demographic and treatment variable of interest. In this analysis, factors that were significant with a *p*-value < 0.05, were carried forward into the multivariable Cox PH model to evaluate survival predictors. A backward stepwise procedure was then executed until all remaining factors in the model were significant (*p* < 0.05). Two-sided statistical tests were used, and all statistical analyses were performed using SPSS, version 23.0 (IBM Corporation, Armonk, NY, USA).

## Results

### Demographic and clinicopathological characteristics

Following ESCC microscopic confirmation, a total of 811 patients who were initially treated with minimally invasive surgery, were identified. 705 patients were enrolled in our study after the application of the exclusion criteria. Table 1 shows the patients’ characteristics. The median survival time for the whole cohort was 56.3 months. 292 patients were confirmed dead, and a total of 413 were censored finally. Of the original group of 705 patients, 678 were alive 1 year after, 591 were alive 2 years after, and 167 were alive 5 years after confirmation of ESCC diagnosis.

**Table 1:** Demographic, tumor, and treatment characteristics of patients after the time of surgery and after 2 year after surgery

Category	Patients from the time of surgery (*n* = 705)		Patients from 2 years after surgery (*n* = 498)	
Age				
≤ 60	399	56.6	290	58.2
> 60	306	43.4	208	41.8
Sex				
Female	173	24.5	124	24.9
Male	532	75.5	374	75.1
Tumor Location				
Upper	63	8.9	39	7.8
Middle	405	57.4	291	58.4
Lower	237	33.6	168	33.7
Tumor Grade				
High	260	36.9	191	38.4
Median	351	49.8	246	49.4
Low	94	13.3	61	12.2
T Stage				
T1	186	26.4	160	32.1
T2	121	17.2	97	19.5
T3	363	51.5	224	45.0
T4a	35	5.0	17	3.4
N Stage				
N0	361	51.2	291	58.4
N1	188	26.7	122	24.5
N2	119	16.9	68	13.7
N3	37	5.2	17	3.4
TNM classification				
I	179	25.4	157	31.5
II	201	28.5	149	29.9
III/IV	325	46.1	192	38.6
Anastomotic Leak				
No	460	90.8	459	92.2
Yes	65	9.2	39	7.8
Pulmonary Infection				
No	553	78.4	402	80.7
Yes	152	21.6	96	19.3
Chylothorax				
No	689	97.7	487	97.8
Yes	16	2.3	11	2.2
Adjuvant Therapy				
No	356	50.5	258	51.8
Chemotherapy	312	44.3	214	43.0
Radiochemotherapy	37	5.2	26	5.2

### Conditional survival

Conditional survival from the time of surgery, and 1, 2, 3, 4, and 5 years after surgery is demonstrated in Table 2. It shows the likelihood of surviving several years given that the patient has already lived a certain amount of time after surgery. For example, the total 5 years survival rate of all stage patients increased and the rates from the time of surgery, and 1, 2, 3, and 4 years after surgery, were 0.55, 0.58, 0.67, 0.77 and 0.88, respectively.

**Table 2:** Probability of surviving to a specific year after having survived a specified number of years after surgery for each stage of disease

Year	Probability of surviving to year after surgery						
	1	2	3	4	5	6	7
**All stage**							
surgery	0.96	0.83	0.72	0.63	0.55	0.52	0.43
1-year after	-	0.86	0.75	0.65	0.58	0.55	0.45
2-year after		-	0.87	0.76	0.67	0.63	0.52
3-year after			-	0.87	0.77	0.73	0.60
4-year after				-	0.88	0.84	0.69
5-year after					-	0.95	0.78
**Stage I**							
surgery	0.98	0.96	0.92	0.85	0.78	0.77	0.63
1-year after	-	0.98	0.93	0.86	0.79	0.78	0.64
2-year after		-	0.95	0.88	0.81	0.80	0.65
3-year after			-	0.93	0.85	0.84	0.69
4-year after				-	0.92	0.90	0.74
5-year after					-	0.98	0.81
**Stage II**							
surgery	0.97	0.87	0.77	0.69	0.61	0.57	0.53
1-year after	-	0.90	0.79	0.71	0.63	0.58	0.55
2-year after		-	0.88	0.80	0.70	0.65	0.61
3-year after			-	0.90	0.80	0.74	0.70
4-year after				-	0.88	0.82	0.77
5-year after					-	0.92	0.87
**Stage III/IV**							
surgery	0.94	0.73	0.59	0.47	0.40	0.38	0.29
1-year after	-	0.78	0.63	0.50	0.43	0.40	0.31
2-year after		-	0.81	0.65	0.55	0.51	0.40
3-year after			-	0.80	0.68	0.64	0.49
4-year after				-	0.85	0.79	0.61
5-year after					-	0.93	0.72

As for the total 7 years survival, patients assigned Stage I disease which was calculated from the time of surgery, had a more unfavorable result (63%) compared with patients assigned Stage II and who have already lived 5 years after surgery (87%). Patients assigned Stage II have a 53% likelihood of surviving 7 years from the time of surgery; whereas patients assigned Stage III/IV have a 72% likelihood of surviving 7 years after they have lived 5 years after surgery (Table 2).

**Fig. 1:**
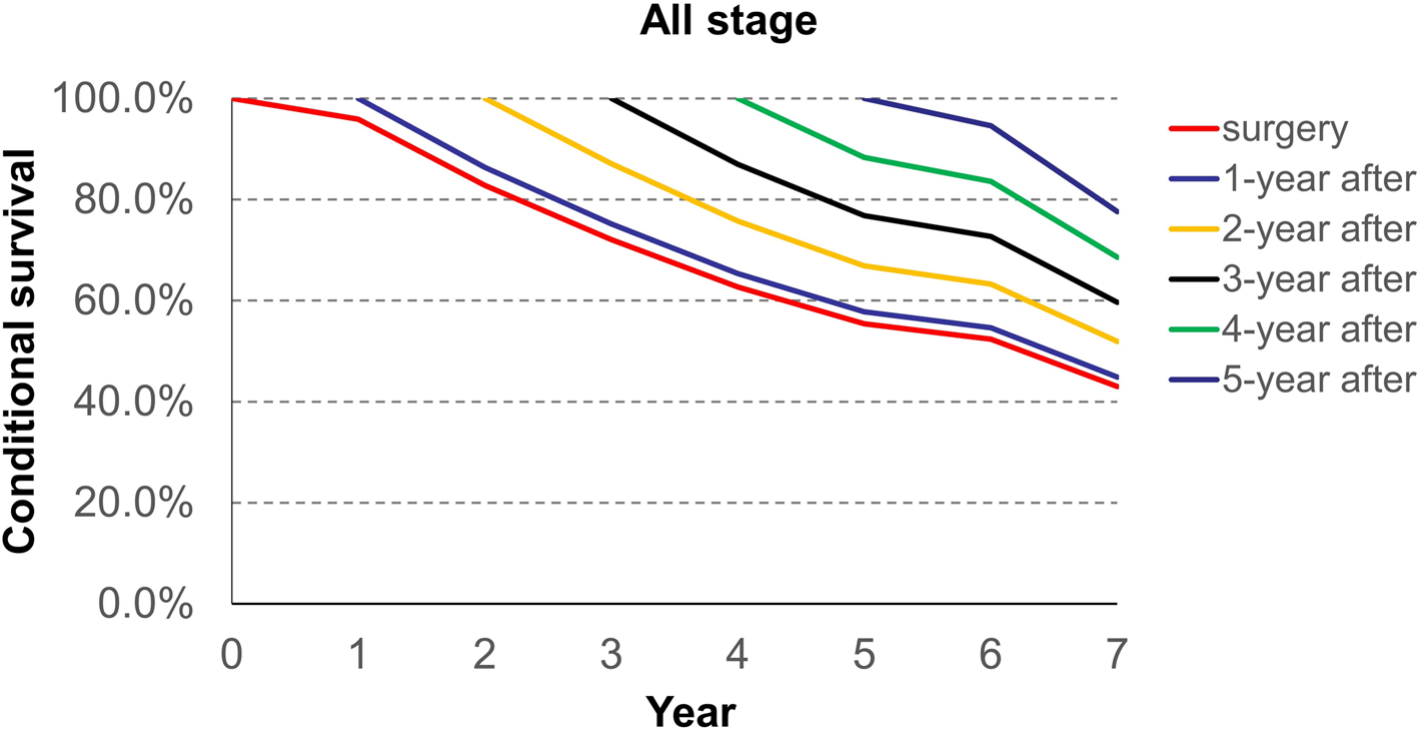
Conditional survival curves for all stage of esophageal squamous cell carcinoma from the time of surgery to 7 years after surgery

The conditional survival rates from the time of surgery to 5 years after surgery for all stages of ESCC patients are shown in Fig. 1. The conditional survival rate between the time of surgery and 1 year after surgery was narrow, then the rate clearly separated from 2 years after surgery to 5 years after surgery.

**Fig. 2:**
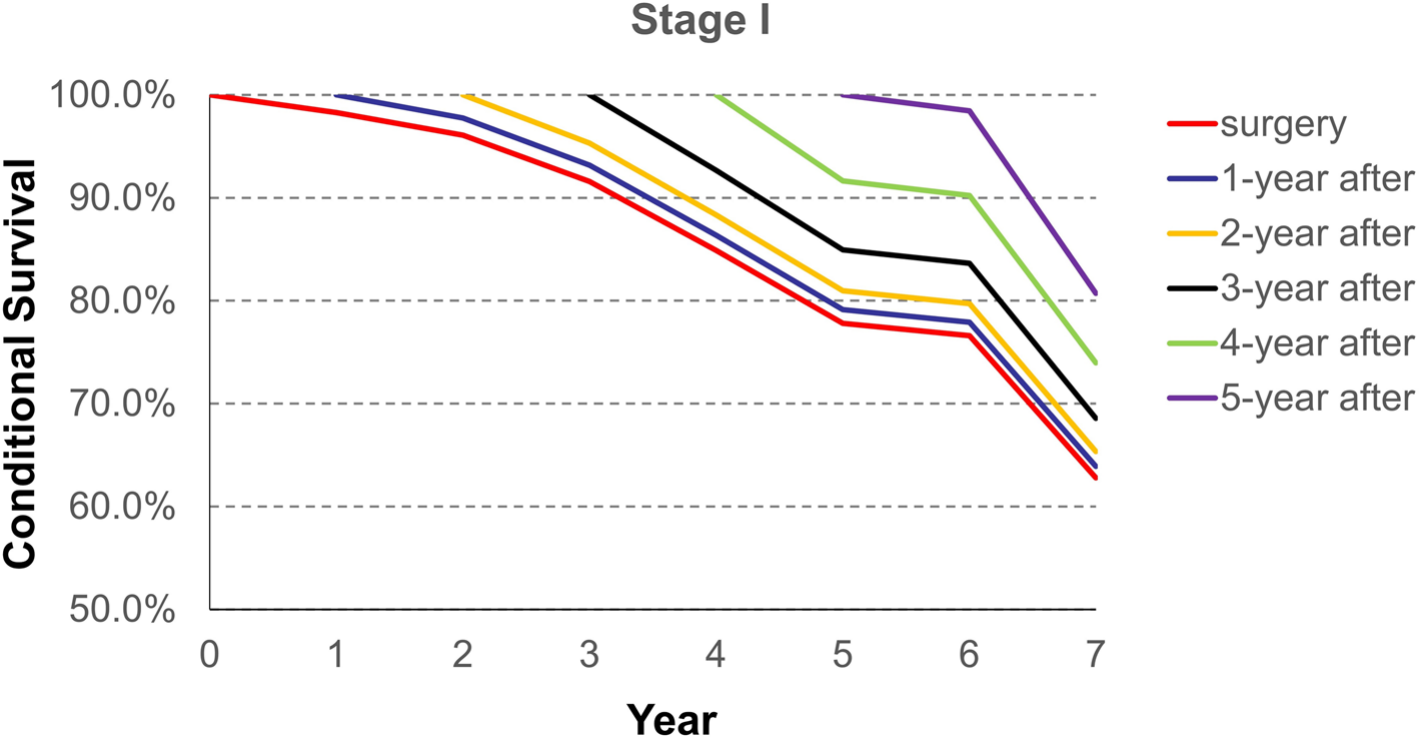
Conditional survival curves for stage I of esophageal squamous cell carcinoma from the time of surgery to 7 years after surgery

However, Fig. 2 shows the conditional survival rates from the time of surgery to 5 years after surgery for stage I of ESCC patients. The results suggest that the likelihood of surviving 5 years is similar with that observed from the time of surgery to 2 years after surgery. However, the survival probability improved from the 3rd year after surgery.

**Fig. 3:**
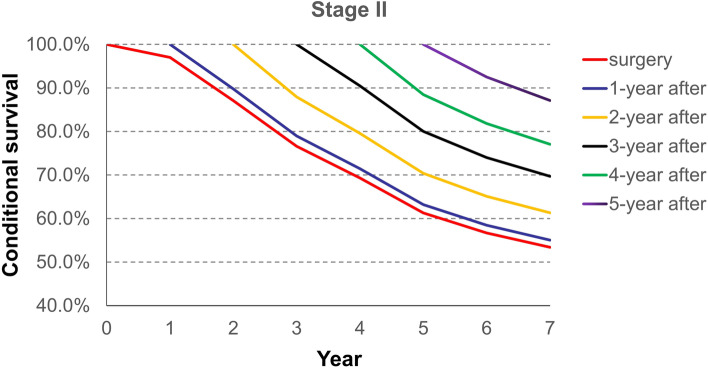
Conditional survival curves for stage II of esophageal squamous cell carcinoma from the time of surgery to 7 years after surgery

**Fig. 4:**
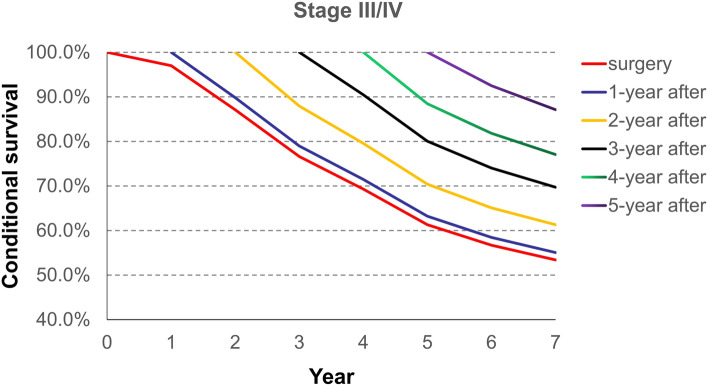
Conditional survival curves for stage III/IV of esophageal squamous cell carcinoma from the time of surgery to 7 years after surgery

Besides, Fig. 3 shows the conditional survival rate for stage II of ESCC patients and Fig. 4 shows the conditional survival rate for stage III/ IV of ESCC patients. The patterns of conditional survival rate for stage II and stage III/IV were similar to those of all stage.

### Factors associated with survival

The above results of the conditional survival analysis indicate that the probability of survival might be different 2 years after surgery. Hence, the characteristics of those patients who already survived 2 years after surgery, are selected (Table 1).

The results of the univariate Cox PH survival model from the time of surgery, tumor grade (*p* = 0.006), T stage (*p* < 0.001), N stage (*p* < 0.001), TNM classification (*p* < 0.001), anastomotic leak (*p* < 0.001) and pulmonary infection (*p* = 0.009), were highly associated with mortality. No significant differences in survival were associated with age, sex, tumor location, chylothorax, and adjuvant therapy (*p* > 0.05). However, the results of the univariate Cox PH survival model from 2 years after surgery, tumor grade (*p* = 0.035), T stage (*p* = 0.001), N stage (*p* < 0.001), TNM classification (*p* < 0.001), and anastomotic leak (*p* = 0.014), were highly associated with mortality. No significant differences in survival were associated with age, sex, tumor location, chylothorax, pulmonary infection, and adjuvant therapy (*p* > 0.05) (Table 3).

**Table 3:** Univariable cox proportional hazards analysis of factors predictive of survival

Category	Patients from the time of surgery		Patients from 2 years after surgery	
	HR (95%CI)	*P*	HR 95%CI)	*P*
Age	1.130(0.897–1.424)	0.301	0.923(0.615–1.384)	0.698
≤ 60				
> 60				
Sex	0.851(0.644–1.125)	0.256	0.763(0.467–1.247)	0.281
Female				
Male				
Tumor Location	0.988(0.816–1.196)	0.902	1.149(0.830–1.593)	0.403
Upper				
Middle				
Lower				
Tumor Grade	1.267(1.071–1.498)	**0.006**	1.351(1.022–1.788)	**0.035**
High				
Median				
Low				
T Stage	1.773(1.542–2.039)	** < 0.001**	1.464(1.178–1.819)	**0.001**
T1				
T2				
T3				
T4a				
N Stage	1.649(1.475–1.844)	** < 0.001**	1.599(1.319–1.939)	** < 0.001**
N0				
N1				
N2				
N3				
TNM Classification	1.981(1.681–2.333)	** < 0.001**	1.649(1.280–2.124)	** < 0.001**
I				
II				
III/IV				
Anastomotic Leak	1.797(1.300–2.485)	** < 0.001**	1.925(1.142–3.245)	**0.014**
No				
Yes				
Pulmonary Infection	1.407(1.088–1.819)	**0.009**	1.11(0.714–1.727)	0.624
No				
Yes				
Chylothorax	0.975(0.460–2.065)	0.947	0.648(0.159–2.629)	0.543
No				
Yes				
Adjuvant Therapy	1.137(0.941–1.375)	0.183	1.262(0.916–1.738)	0.154
No				
Chemotherapy				
Radiochemotherapy				

The results of the multivariable Cox PH survival model from the time of surgery, T stage [hazard ratio (HR): 1.507, 95% confidence intervals (95%CI): 1.298–1.748; *p* < 0.001], N stage (HR: 1.464, 95%CI: 1.297–1.653; *p* < 0.001), and anastomotic leak (HR: 1.472, 95%CI: 1.057–2.050; *p* = 0.022), were significantly associated with survival, while tumor grading and TNM classification were not. However, the results of the multivariable Cox PH survival model from 2 years after surgery, N stage (HR: 1.499, 95%CI: 1.218–1.845; *p* < 0.001), and anastomotic leak (HR: 1.804, 95%CI: 1.053–3.094; *p* = 0.032), were significantly associated with survival, while tumor grading, T stage and TNM classifications were not. Table 4 displays the results of the cox PH survival model.

**Table 4:** Multivariable cox proportional hazards analysis of factors predictive of survival

Category	Patients from the time of surgery		Patients from 2 years after surgery	
	HR (95%CI)	*P*	HR 95%CI)	*P*
Tumor Grade	1.134(0.960–1.339)	0.138	1.274(0.962–1.687)	0.091
High				
Median				
Low				
T Stage	1.507(1.298–1.748)	** < 0.001**	1.220(0.967–1.538)	0.094
T1	ref			
T2	1.404(0.891–2.210)	0.143		
T3	2.409(1.662–3.492)	** < 0.001**		
T4a	2.784(1.618–4.789)	** < 0.001**		
N Stage	1.464(1.297–1.653)	** < 0.001**	1.499(1.218–1.845)	** < 0.001**
N0	ref		ref	
N1	1.484(1.100–2.001)	**0.010**	1.194(0.719–1.982)	0.493
N2	2.300(1.681–3.146)	** < 0.001**	3.335(2.064–5.389)	** < 0.001**
N3	2.950(1.905–4.457)	** < 0.001**	2.608(1.166–5.835)	**0.020**
TNM Classification	1.011(0.744–1.374)	0.944	0.810(0.477–1.375)	0.435
I				
II				
III/IV				
Anastomotic Leak	1.472(1.057–2.050)	**0.022**	1.804(1.053–3.094)	**0.032**
No	ref		ref	
Yes	1.508(1.081–2.104)	0.016	2.042(1.206–3.460)	**0.008**
Pulmonary Infection	1.122(0.856–1.471)	0.405		
No				
Yes				

## Discussion

As ESCC patients survive from the time of surgery, they and their counsels may be focus on whether the likelihood of surviving a certain amount of time (e.g., 5 years), rises as the patients ongoing survive. Traditional survival estimates that were computed from the time of diagnosis, are less significant and may even be misleading for patients who have already lived for a specified number of years after cancer diagnosis. This is due to timely changes in the prognosis of each individual patient.

Analysis of conditional survival offers additional perspectives on cancer survival after radical surgery [16]. For example, the likelihood of surviving 5 years after surgery for all patients is 55%; the likelihood of surviving 3 years after already lived 2 years is 67%; the likelihood of surviving 1 year after already lived 4 years is still 88% (Fig. 1). It seems that the likelihood of surviving slightly increases as the patient continue to survive from the time of surgery.

The trend of survival rates for each stage, except for stage I disease, from 2 years after surgery later, are better than traditional survival, regardless of the amount of time lived. The conditional survival rate for all patients’ stages starting at year 1 after surgery was similar to traditional survival. However, from year 2 to year 5 after surgery, the conditional survival rate for all stages, was better than traditional survival. The reasons of the difference between the conditional overall survival of stage I esophageal cancer and other stages are as follows: 1) It is well known that the recurrence rate of patients with stage I esophageal carcinoma is lower than that of other stages; 2) Those patients did not need postoperative adjuvant therapies, including radiotherapy, chemotherapy, chemoradiotherapy), which would prevent side effects; and 3) Although postoperative adjuvant therapies can kill the underlying tumor cells, that can also destroy the patients’ immunity which may lead to adverse oncological outcomes.

Although no changes were observed in stage II and III/IV, the survival of stage I suddenly dropped after six years. This was an interesting phenomenon and several reasons can explain this status. When compared with stage II-IV esophageal cancer patients, those patients with stage I esophageal cancer did not need to receive postoperative adjuvant therapies, according to many different treatment guidelines of esophageal cancer. In our survey, a large proportion of those patients believed that they had been cured, and their attitude to follow-ups was not positive, especially 5 years after the surgery. Therefore, postoperative recurrence and metastasis could not be detected in time. After this study, we strongly recommended that all patients must receive routine postoperative follow-ups after the surgery.

In this study, the conditional survival was calculated by the Kaplan–Meier method. The probability of conditional survival from the time of surgery reflected traditional survival [5]. To a certain extent, this indicates that the relatively stable effect of traditional survival on survival. It is significant that traditional survival also lost its capability to affect patient overall survival after 2 years of survival, as observed at the time of original diagnosis, and regardless of the clinical status. That is to say, those surviving beyond 2 years, are possibly biologically diverse from those in the initial cohort and many other aspects may affect survival from the time of surgery.

The T stage is an independent prognostic factor for the time to surgery model but not for the model from 2 years after surgery. Anatomically, the esophagus is different from other alimentary canals. There is no serous membrane in the esophagus, and therefore, the T stage of esophageal carcinoma is difficult to evaluate. The ultrasonic gastroscope can not pass through to correctly examine for intraluminal-type esophageal tumors. These patients’ preoperative T stage might be downgraded resulting in a residual tumor during the operation or an local recurrence after the operation. Patients with a high T stage would receive postoperative therapies, such as radiotherapy or chemoradiotherapy, which could kill the residual cancer. Hence, the risk of recurrence was significantly reduced in the first 2 years and the T Stage played a key role during this period. However, the local recurrence and distant metastasis might be the main reasons for advanced esophageal disease in patients after 3 years from the surgery. During this period, many factors were involved and the T stage played a small role.

Previous studies indicated the use of prognostic factors were related to the survival outcomes, especially those variables that evaluated at the time of surgery might result in incorrectly results [17]. The detection of these factors is essential to patient supervision. In this study, we found that N stage and anastomotic leak were associated with survival regardless of the time, while T stage was only associated with survival within 2 years after surgery. This is critical to our follow-up strategy, and we recommend that patients with anastomotic leakage and higher T and N stages should be strictly screened to reduce poor survival within the 2 years after surgery. Afterward, patients should be screened for those patients with anastomotic leak and a high N stage.

After esophagectomy, several important early complications including pulmonary infection, anastomotic leakage, and bleeding. For instance, pulmonary infection was an important cause of postoperative death. In our center, several measures had been taken to prevent pulmonary infection and the incidence of pulmonary infection is very low. These measures included: 1) A single-lumen endotracheal intubation with two-lung ventilation was used to reduce iatrogenic atelectasis; 2) Bilateral recurrent laryngeal nerve protection methods were used to avoid the injury of the vocal cords; and 3) A fiberoptic bronchoscope was used to assist in expectoration. However, the effect of pulmonary infection was transitory for a patient. When the patients recovered, the influence of pulmonary infection was over.

The anastomotic leakage was a very difficult problem. First, the patients with anastomotic leakage might suffer from malnutrition for a long period of time. Second, anastomotic stricture was often accompanied by an anastomotic leakage and the systemic malnutrition effects might be long-lasting. Third, the anastomotic leakage might interfere with the schedule of postoperative adjuvant therapy. In short, the anastomotic leakage had a great influence on the patients’ oncological outcome.

There are several limitations in our study. First, this is a small-scale and single center clinical study, which needs a large sample and multiple-centers further external validation. Second, we only focused on the overall survival, the other survival indexes such as disease-free survival, disease-specific survival, were not included. Third, the results are derived from the ESCC patients, which may not be suit for those other pathologic type esophageal cancer.

## Conclusion

ESCC patients who received a minimally invasive surgery showed a better conditional survival 2 years after surgery. These data provide a powerful perspective on conditional survival among long term ESCC survivors that may be beneficial in patients’ therapy. We suggest that patients with anastomotic leakage and higher T and N stages should be strictly screened at various times, and that conditional survival should be used as a prognostic tool for patients’ evaluation.

## Data Availability

The datasets used and/or analyzed during the current study are available from the corresponding author on reasonable request.

## Abbreviations

EA: Esophageal adenocarcinoma
ESCC: Esophageal squamous cell carcinoma
SEER: Surveillance, Epidemiology, and End Results database
EMR: Electronic medical records
OS: Overall survival
CS: Conditional survival
HR: Hazard ratio
95%CI: 95% Confidence intervals
